# Targeted next-generation sequencing for antimicrobial resistance detection in ventilator-associated pneumonia

**DOI:** 10.3389/fcimb.2025.1526087

**Published:** 2025-01-31

**Authors:** Yuting Li, Yanfang Jiang, Hao Liu, Yao Fu, Junying Lu, Hongyan Li, Lulu Sheng, Dejian Gu, Dong Zhang

**Affiliations:** ^1^ Department of Critical Care Medicine, The First Hospital of Jilin University, Changchun, China; ^2^ Genetic Diagnosis Center, The First Hospital of Jilin University, Changchun, China; ^3^ Department of Medicine, GenePlus-Beijing, Beijing, China

**Keywords:** targeted next-generation sequencing, ventilator-associated pneumonia, pathogen, antimicrobial resistance, intensive care unit

## Abstract

**Background:**

Ventilator-associated pneumonia (VAP) carries a high mortality rate in the intensive care units (ICUs) due to its widespread drug resistance. Traditional microbial cultures limited by speed and sensitivity, are often unable to help clinicians make effective diagnosis and treatment. Therefore, there is an urgent need for a rapid and accurate test that can identify both pathogens and their antimicrobial resistance (AMR) to improve the prognosis of patients with VAP.

**Study design:**

We analyzed samples from ICU patients with suspected VAP using both microbial tests and targeted next-generation sequencing (tNGS), comparing the results of tNGS pathogen and AMR detection against microbial culture and antimicrobial susceptibility testing (AST).

**Results:**

Involving 199 patients with suspected VAP, tNGS showed a sensitivity rate of 98.98% for pathogen identification. While the sensitivity rate of microbial culture was just 66.84%. Additionally, tNGS performed almost half the turnaround time of microbial culture (1.66 days vs 3.00 days). For AMR, the overall consistency between AST and tNGS was 79.31%. The great performance particularly exhibited for *Acinetobacter baumannii* carbapenem-penicillin-cephamycin resistance.

**Conclusion:**

tNGS excels in identifying pathogens and AMR. Its rapid workflow makes it ideal for managing critically ill patients, enhancing treatment precision, and reducing antibiotic misuse.

## 1 Introduction

Ventilator-associated pneumonia (VAP) represents a significant complication in critically ill patients, accounting for substantial mortality rates within intensive care units (ICUs) globally (Wicky et al., 2023; Vacheron et al., 2022). VAP, a common nosocomial infection, increases hospital stays and healthcare costs. In addition, polymicrobial nature and resistance patterns complicate the problem, highlighting the need for rapid, accurate diagnostics (Cabrera-Tejada et al., 2023; Duszynska et al., 2022; Stewart et al., 2021).

Traditional diagnostic methods, predominantly reliant on microbial cultures, are hampered by lengthy processing times and limited sensitivity, often leading to delayed or empirical treatment (Peri et al., 2022; Shin et al., 2021).

The advent of targeted next-generation sequencing (tNGS) heralds a transformative era in infectious disease diagnostics, offering the ability to detect polymicrobial infections and rapid turnaround time, alongside antimicrobial resistance (AMR) genes for guiding precision therapy (Li et al., 2022). Recently, some studies have explored the application of tNGS in clinical infection detection with diagnostic accuracy of 65.6%-76.9%, underscoring its vast potential for future diagnostic practices (Gaston et al., 2022; Wu et al., 2023).

Despite the promising attributes of tNGS, its application in diagnosing pathogens and antimicrobial resistance of ICU patients remains underexplored. This study aims to assess the performance of tNGS in the identification of pathogens and their AMR genes in patients with suspected VAP. By bridging this research gap, the study endeavors to contribute to the refinement of diagnostic strategies for VAP, potentially overcoming cost or accessibility barriers, facilitating the reduction in diagnostic delays, enhancing the precision of antimicrobial therapy, and ultimately improving patient outcomes in critical care settings.

## 2 Materials and methods

### 2.1 Study design and population

In this retrospective study, we enrolled adult patients (aged 18 years or older) admitted to the ICU of the First Hospital of Jilin University between May 2023 and March 2024. Eligibility criteria included having (1) sufficient lower respiratory tract samples, (2) results from microbial cultures, (3) available clinical information. Lower respiratory tract samples were collected within 12 hours of patients being suspected of VAP. Suspected VAP was defined as pneumonia occurring after receiving mechanical ventilation lasting 48 hours or within 48 hours of withdrawal of mechanical ventilation. Pneumonia was defined as meeting all two of the following criteria: (1) at least one compatible symptom, such as new-onset fever, cough, or dyspnea, (2) new-onset radiological findings on chest images. For each patient, a comprehensive clinical diagnosis result of infectious pathogens was collected. It was determined by at least two clinicians based on a combination of conventional microbiological tests and the clinical presentation of patient. Conventional microbiological tests included culture, PCR, G/GM test, and antigen/antibody test. All samples were performed conventional microbiological tests in parallel with tNGS.

Antimicrobial susceptibility testing (AST) for the positive microbial culture were assessed on the Vitek-2 system (bioMérieux, Marcy l’Etoile, France). The susceptibility testing results were interpreted according to the criteria recommended by the Clinical and Laboratory Standards Institute (CLSI, 2022).

### 2.2 Targeted next-generation sequencing

Lower respiratory tract samples were collected for analysis, including bronchoalveolar lavage fluid (BALF) and sputum. BALF samples were obtained exclusively from the middle segment. Sputum samples were collected from patients’ first deep cough episodes in the early morning, following mouth rinsing with sterile saline 2-3 times (Li et al., 2023; Zhang et al., 2024). A mixture consisting of lysis buffer, protease K, and binding buffer was promptly added to these samples within a grinding tube. The samples underwent mechanical lysis for 30 seconds using a shock breaker, ensuring thorough disruption. Subsequent to this, DNA and RNA were co-extracted using the VAMNE Magnetic Pathogen DNA/RNA Extraction Kit (Vazyme, Nanjing, China), as specified by the manufacturer’s protocol. Quantification of the extracted nucleic acids was accurately achieved using a Qubit 3.0 fluorometer (Invitrogen, California, USA), with the employment of high sensitivity assay kits (Invitrogen, California, USA) for both double-stranded DNA and RNA, ensuring precise measurement of nucleic acid concentrations.

To synthesize complementary DNA and prepare the sequencing library, we employed the HieffNGS^®^C37P4 OnePot cDNA & gDNA Library Prep Kit (Yeasen, Shanghai, China), following the protocols provided by the manufacturer. Enrichment of the target sequences was conducted by incubating the samples with GenePlus probes (GenePlus, Beijing, China) for approximately four hours. This step was followed by the amplification of the captured products through an 18-cycle polymerase chain reaction (98°C 15 s, 60°C 30 s, 72°C 30 s). Subsequently, the amplified products underwent processing to generate DNA nanoballs by One-Step DNB Preparation Kit (GenePlus, Beijing, China).

Sequencing was performed using the Gene^+^Seq-100 platform (GenePlus, Beijing, China) with 100-bp single-end reads. The procedure aimed for a sequencing depth of 5 million reads, to ensure comprehensive coverage of the targeted regions.

In the sequencing data analysis, we employed GenePlus’s proprietary data analysis solution (GenePlus, Beijing, China) for initial processing. This entailed the elimination of sequences of low quality, the removal of residual adapters, and the exclusion of reads below a threshold length. Furthermore, sequences identified as microbial ribosomal RNA or human genomic material were filtered out. Subsequently, the processed data was aligned and annotated against an extensive database of pathogenic microorganisms and AMR using BLAST for sequence comparison. Reads that matched the target capture regions were designated as target reads and normalized on a reads per million (RPM) basis, enabling quantitative analyses. This process yielded a comprehensive pathogen and AMR profile within each sample. To minimize the introduction of environmental or experimental contaminants, an additional blank control sample was included in each sequencing run. This practice serves to identify and exclude reads that may have arisen from contamination. Subsequently, clinical significant microbes were defined by the criteria of Peng et al. (Peng et al., 2021).

### 2.3 Statistical analysis

Scoring structure for tNGS comparisons to clinical results is referenced from Karius (Blauwkamp et al., 2019). Paired nonparametric variables were compared using the Wilcoxon test and Friedman test. Unpaired nonparametric variables were compared using the Mann-Whitney test. All tests were two-tailed and significance was set at 5%. In the calculation of diagnostic performance, consistency, sensitivity, specificity, positive predictive value (PPV), negative predictive value (NPV), positivity, and F_1_ score were computed using the standard proportion formula, and the 95% confidence intervals (CI) for these proportions were determined using the Wilson method. All figures were drawn using R version 4.3.1 and GraphPad Prism version 9.5.0 for Windows (GraphPad Software LLC., San Diego, CA, USA). All analyses were performed with SPSS version 26.0 for Windows (SPSS Inc., Chicago, Illinois, USA).

## 3 Results

### 3.1 Study population

**Figure 1:**
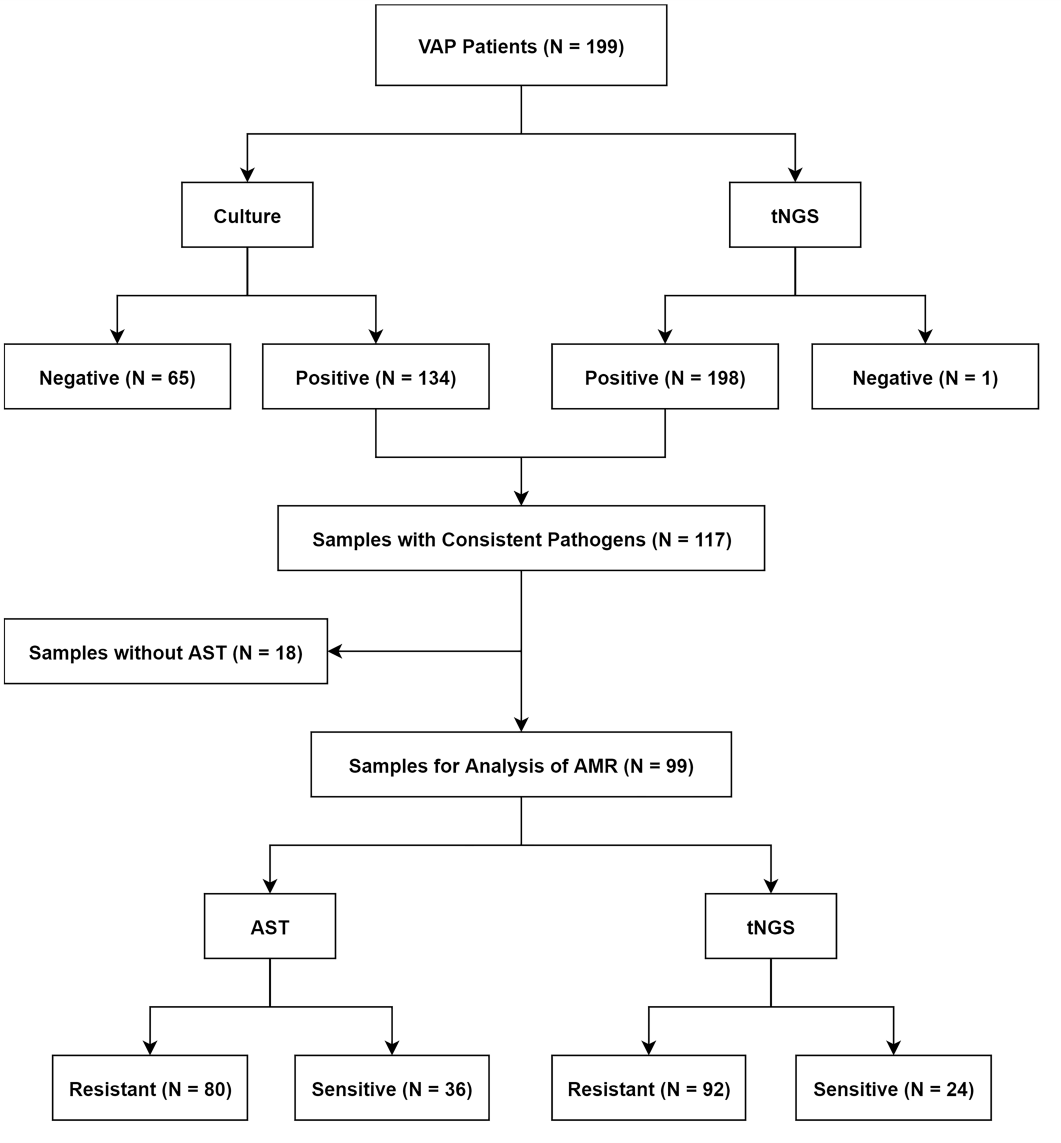
Workflow for the screening of research cases.

In this study, a cohort of 199 patients suspected of VAP was recruited from the ICU at the First Hospital of Jilin University (**Supplementary Table 1**). Based on the comprehensive clinical diagnosis, 196 patients were diagnosed with VAP. Among all samples, 134 tested positive using microbial culture, while 198 tested positives using tNGS. 117 Samples were obtained for pathogens identification using both microbial culture and tNGS. Of these, 18 samples were excluded due to the absence of AST. The remaining 99 samples underwent analysis for AMR consistency between AST and tNGS results (**Figure 1**).

### 3.2 Diagnostic performance of tNGS in pathogen identification of patients with VAP

In the analysis of 199 samples, tNGS demonstrated a consistency rate of 98.49% (196/199) and a sensitivity rate of 98.98% (194/196) when compared to the comprehensive clinical diagnosis for pathogen identification. In bacteria, tNGS also showed excellent performance, with a consistency rate of 91.46% and a sensitivity rate of 96.99%, which is the basis for accurate identification of AMR (**Table 1**). Common pathogens included *Acinetobacter baumannii*, *Klebsiella pneumoniae*, and *Pseudomonas aeruginosa* (**Supplementary Figure 2**). While microbial culture just performed a consistency rate of 66.83% (133/199) and a sensitivity rate of 66.84% (131/196), which significantly lower than tNGS (**Table 1**). Additionally, tNGS demonstrated a notably shorter turnaround time (P < 0.05). From sample collection to the final report, the entire process took just 1.66 (1.63 - 1.69) days with tNGS, nearly half of the time required for microbial culture (3.00 days) (**Supplementary Figure 1**).

**Table 1:** Performance of tNGS and microbial culture in pathogen identification compared to the comprehensive clinical diagnosis.

Technology	Type	Consistency	Sensitivity	Specificity	PPV	NPV	F_1_
tNGS	Bacteria	91.46% (86.75% - 94.60%)	96.99% (93.14% - 98.71%)	63.64% (46.62% - 77.81%)	93.06% (88.27% - 95.99%)	80.77% (62.12% - 91.49%)	0.95 (0.91 - 0.97)
	Fungi	79.40% (73.25% - 84.43%)	100.00% (82.41% - 100.00%)	77.35% (70.72% - 82.84%)	30.51% (20.25% - 43.15%)	100.00% (97.33% - 100.00%)	0.47 (0.33 - 0.60)
	Virus	38.19% (31.72% - 45.10%)	100.00% (87.13% - 100.00%)	28.90% (22.66% - 36.06%)	17.45% (12.20% - 24.34%)	100.00% (92.86% - 100.00%)	0.30 (0.21 - 0.39)
	All	98.49% (95.66% - 99.49%)	98.98% (96.36% - 99.72%)	66.67% (20.77% - 93.85%)	99.49% (97.15% - 99.91%)	50.00% (15.00% - 85.00%)	0.99 (0.97 - 1.00)
Culture	Bacteria	77.89% (71.63% - 83.10%)	74.70% (67.58% - 80.70%)	93.94% (80.39% - 98.32%)	98.41% (94.40% - 99.56%)	42.47% (31.78% - 53.90%)	0.85 (0.79 - 0.89)
	Fungi	95.48% (91.63% - 97.60%)	66.67% (43.75% - 83.72%)	98.34% (95.24% - 99.43%)	80.00% (54.81% - 92.95%)	96.74% (93.07% - 98.50%)	0.73 (0.49 - 0.88)
	All	66.83% (60.03% - 73.00%)	66.84% (59.98% - 73.05%)	66.67% (20.77% - 93.85%)	99.24% (95.83% - 99.87%)	2.99% (0.82% - 10.25%)	0.80 (0.74 - 0.84)

### 3.3 Analysis the AMR consistency between AST and tNGS

In 99 samples with both AST and AMR tNGS results, 116 bacteria were identified (**Supplementary Figure 3**). In AMR detection, with the exception of aminoglycoside, which has a consistency rate of 60.34% (70/116), all other AMR exceed 70%, especially lincomycin (98.28%, 114/116). However, in terms of sensitivity, tNGS showed 60.00% (3/5) in lincomycin and 42.00% (21/50) in tetracycline, which means it missed some AMR compared to AST. While in carbapenem-penicillin-cephamycin and aminoglycoside, tNGS performed high sensitivity, both exceeding 80%. In terms of specificity, tNGS showed 96.97% (64/66) in tetracycline and 100.00% (111/111) in lincomycin. But it showed specificity rates of 54.76% (23/42) in carbapenem-penicillin-cephamycin and 42.42% (28/66) in aminoglycoside, which means tNGS identified some additional AMR. Overall, tNGS demonstrated a consistency rate of 79.31% (92/116) compared to AST (**Table 2**).

**Table 2:** Performance of tNGS in AMR identification.

AMR	Consistency	Sensitivity	Specificity	PPV	NPV	F_1_
Carbapenem, penicillin, cephamycin	76.72% (68.25% - 83.48%)	89.19% (80.09% - 94.42%)	54.76% (39.95% - 68.78%)	77.65% (67.71% - 85.20%)	74.19% (56.75% - 86.30%)	0.83 (0.73 - 0.90)
Aminoglycoside	60.34% (51.25% - 68.78%)	84.00% (71.49% - 91.66%)	42.42% (31.24% - 54.44%)	52.50% (41.70% - 63.08%)	77.78% (61.91% - 88.28%)	0.65 (0.53 - 0.75)
Sulfonamide	75.00% (66.40% - 81.99%)	79.17% (65.74% - 88.27%)	72.06% (60.44% - 81.32%)	66.67% (53.72% - 77.51%)	83.05% (71.54% - 90.52%)	0.73 (0.59 - 0.83)
Tetracycline	73.28% (64.57% - 80.49%)	42.00% (29.37% - 55.77%)	96.97% (89.61% - 99.17%)	91.30% (73.20% - 97.58%)	68.82% (58.81% - 77.33%)	0.58 (0.42 - 0.71)
Lincomycin	98.28% (93.93% - 99.53%)	60.00% (23.07% - 88.24%)	100.00% (96.65% - 100.00%)	100.00% (43.85% - 100.00%)	98.23% (93.78% - 99.51%)	0.75 (0.30 - 0.94)
All	79.31% (71.06% - 85.68%)	92.50% (84.59% - 96.52%)	50.00% (34.47% - 65.53%)	80.43% (71.18% - 87.25%)	75.00% (55.10% - 88.00%)	0.86 (0.77 - 0.92)

**Figure 2:**
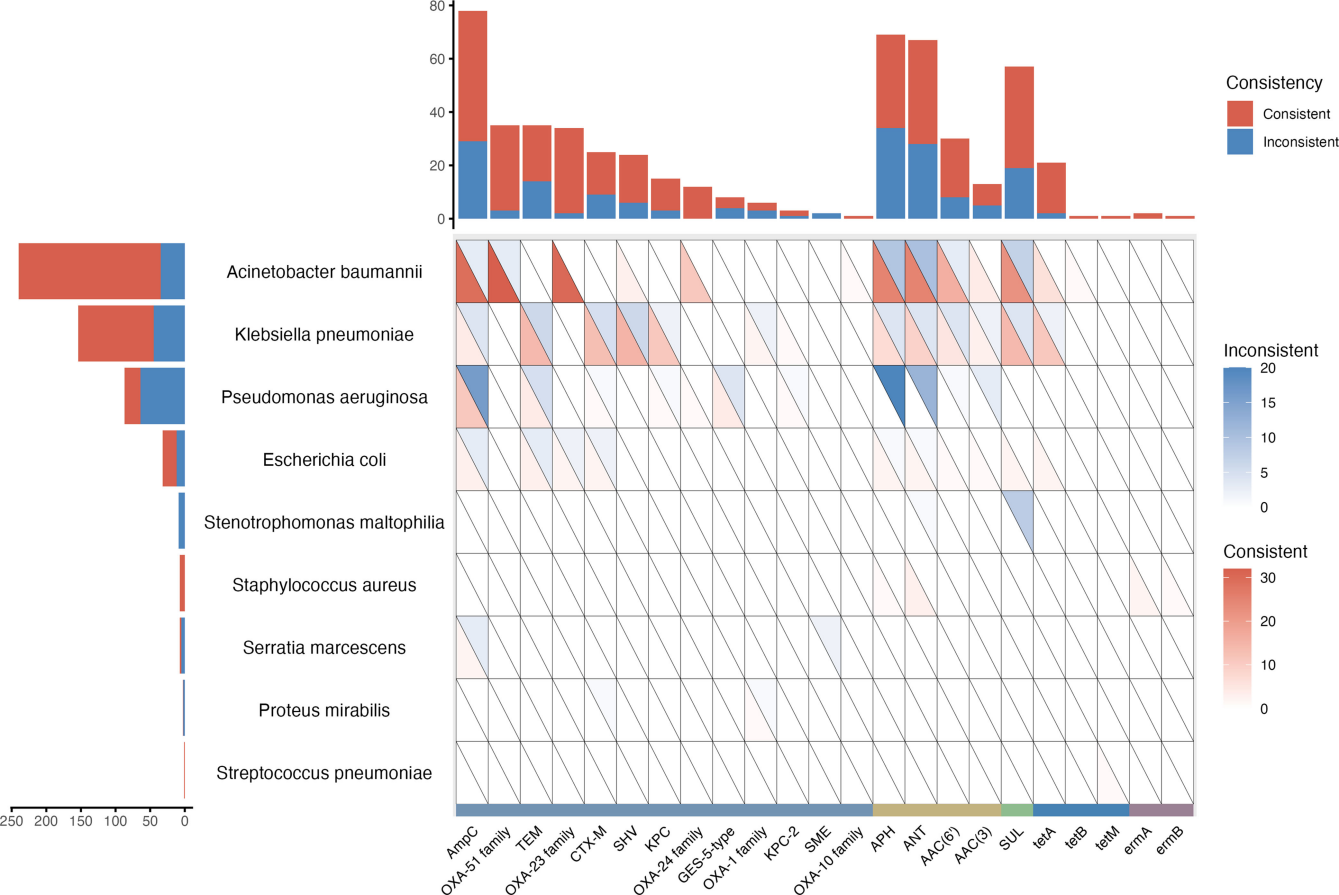
AMR genes reported by tNGS and the consistency with AST.

The AMR consistency between AST and tNGS was further analyzed. At the genetic level, common AMR genes identified by tNGS included *ampC*, *APH*, *ANT*, and *sul*, mainly related to *A. baumannii* and *K. pneumoniae*. In *A. baumannii*, frequently detected genes related to carbapenem-penicillin-cephamycin resistance, such as *ampC*, *blaOXA-51*, and *blaOXA-23* families, showed PPV of 90.63% (29/32), 91.43% (32/35), and 100.00% (30/30), respectively, when compared to AST. In *K. pneumoniae*, the common genes linked to carbapenem-penicillin-cephamycin resistance included *blaTEM*, *blaCTX-M*, *blaSHV*, and *blaKPC*, with PPV of 60.87% (14/23), 61.90% (13/21), 62.50% (15/24), and 68.75% (11/16), respectively. For aminoglycoside resistance, the commonly identified genes *APH* and *ANT* showed PPV of 71.43% (25/35) and 69.44% (25/36) in *A. baumannii*, and 53.85% (7/13) and 60.00% (9/15) in *K. pneumoniae*. The sulfonamide resistance gene *sul* was detected with a PPV of 73.33% (22/30) in *A. baumannii* and 73.68% (14/19) in *K. pneumoniae*. However, in *P. aeruginosa*, the PPV was just 26.44%, likely due to the additional AMR genes detection of tNGS (**Figure 2**).

### 3.4 Analysis the reason for additional detected AMR

**Figure 3:**
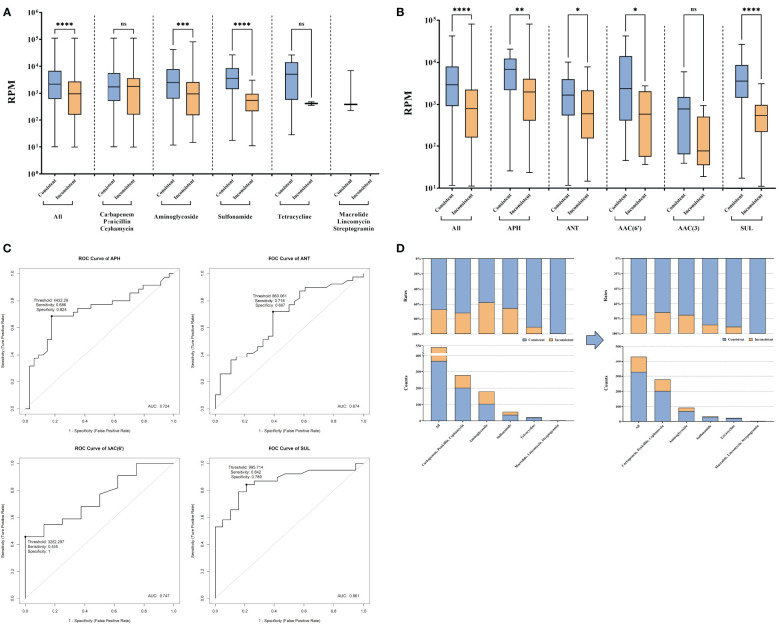
AMR genes exclusively reported by tNGS compared to AST. **(A)** Reads of AMR genes consistent and inconsistent with AST in AMR types. **(B)** Reads of aminoglycoside and sulfonamide resistance genes. **(C)** Exploration of new reporting thresholds for *APH*, *ANT*, *AAC(6')*, and *SUL*. **(D)** Improvement of the consistency under new reporting thresholds. ns and * represent the p-value for the significance test. ns means p-value > 0.05, * means p-value < 0.05, ** means p-value < 0.01, *** means p-value < 0.001, **** means p-value < 0.0001.

To investigate the underlying cause for the additional detected AMR and address the associated challenges, the RPM differences between consistent AMR and inconsistent AMR were further analyzed. In the comparison, a significantly lower RPM of AMR genes identified exclusively by tNGS was observed, especially those related to aminoglycoside and sulfonamide (P < 0.05) (**Figure 3A**). For AMR related to aminoglycoside and sulfonamide, more detailed analyses showed the RPM difference was attributed to *APH*, *ANT*, *AAC(6’)*, and *sul* (P < 0.05) (**Figure 3B**). To enhance the AMR consistency between AST and tNGS, using receiver operating characteristic (ROC) analysis, the optimal report thresholds for these four AMR genes were established (**Figure 3C**). After applying these refined thresholds to tNGS results, an enhancement in the AMR gene consistency rate was observed, which increased from 68.15% (368/540) to 75.93% (328/432) (**Figure 3D**).

### 3.5 tNGS missed some AMR compared to AST

**Figure 4:**
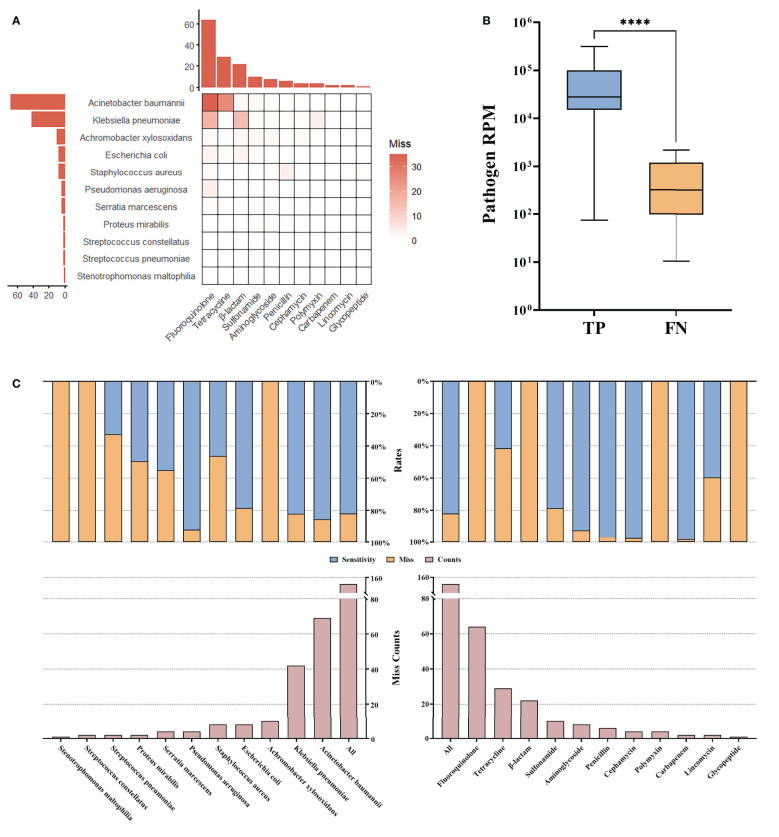
AMR missed by tNGS. **(A)** Miss compared to AST. **(B)** Reads of pathogens for which miss AMR. **(C)** Statistical rates. **** means the p-value for the significance test < 0.0001.

In some bacteria with AMR, anticipated AMR genes were not found by tNGS, primarily including fluoroquinolone or tetracycline resistant *A. baumannii*, and fluoroquinolone or β-lactam (except carbapenem, penicillin, and cephamycin) resistant *K. pneumoniae* (**Figure 4A**). A deeper examination revealed that these bacteria displayed significantly lower RPM, suggesting a correlation between the lower pathogen abundance and the consequent reduced detectability of AMR. This indicates that the limitations in identifying AMR genes via tNGS could be attributed to the low abundance of the carrier pathogens, leading to insufficient genomic representation of the AMR genes (**Figure 4B**). The overall miss rate was noted to be 17.67% (152/860). Specifically, the miss rates for detecting AMR in *A. baumannii* and *K. pneumoniae* were 14.20% (69/486) and 17.50% (42/240), respectively. Additionally, tNGS did not identify any AMR gene associated with fluoroquinolones and β-lactam (except carbapenem, penicillin, and cephamycin), resulting in 100.00% (64/64, 22/22) miss rates. Besides, a 58.00% (29/50) miss rate was reported for tetracycline resistance (**Figure 4C**). These results highlight the potential limitations of tNGS in the precise identification of AMR across specific antibiotic categories, notably fluoroquinolones, β-lactam (except carbapenem, penicillin, and cephamycin), and tetracyclines.

## 4 Discussion

In this study, we address the critical challenge posed by VAP, a hospital-acquired infection notorious for its high mortality rates. The pathogens responsible for VAP are often resistant to multiple drugs, necessitating precise and targeted therapy to effectively control the infection and reduce fatalities (Denis et al., 2019; Rezia and Vijendra, 2023). Conventional diagnostic methods, such as microbial cultures and AST, are time-consuming and have low sensitivity, making them inadequate for the urgent diagnostic needs of patients with VAP. However, tNGS as a technique of high potential, has not been extensively referenced for application in the diagnosis and treatment of patients with VAP until now (Chen et al., 2024). Our research fills this significant gap by thoroughly evaluating the performance of tNGS in detecting pathogens and resistance genes in patients with VAP. By comparing these findings with those of traditional clinical tests and delving into the differences, our study offers comprehensive insights and valuable references for the future clinical application of tNGS in managing VAP, potentially transforming the approach to diagnosis and treatment in these critically ill patients.

Our research demonstrated the superior capability of tNGS in detecting pathogens and AMR genes in patients with VAP. In pathogen identification, tNGS exhibited high consistency and sensitivity compared to microbial cultures, aligning with findings from previous studies that highlight the limitations of traditional methods (Gan et al., 2024). Notably, the median turnaround time for tNGS in our study was 1.66 days, nearly half the time required for microbial cultures, with potential for further reduction to as little as 18 hours (Zhang et al., 2024). These findings underscore the transformative potential of tNGS in accelerating diagnostic workflows for critically ill patients. For AMR detection, tNGS demonstrated substantial consistency with AST, particularly in identifying resistance to carbapenem, penicillin, cephalosporin, aminoglycoside, and sulfonamide. Importantly, tNGS accurately identified resistance to pathogens classified as critical by the WHO’s 2024 updated bacterial priority pathogens list, including carbapenem-resistant *A. baumannii*, third-generation cephalosporin-resistant enterobacterales, and carbapenem-resistant enterobacterales. This highlights the pivotal role of tNGS in addressing global health challenges posed by drug-resistant infections.

Although similar studies across diverse patient cohorts have reported slightly lower pathogen identification performance compared to our findings, the results remain impressively robust. For instance, a study focusing on pediatric patients with lower respiratory tract infections revealed a sensitivity rate of 84.40% (Lin et al., 2023), and another investigation into adult patients with periprosthetic joint infections noted a sensitivity rate of 88.37% (Huang et al., 2023). These variations underscore the influence of patient cohort characteristics on the performance of tNGS in pathogen detection, highlighting its adaptability and robustness across diverse clinical scenarios. For antimicrobial resistance detection, the performance of tNGS also surpassed that observed in previous reports. For example, a study on pediatric patients with severe pneumonia revealed that the sensitivity rates of metagenomic next-generation sequencing (mNGS) for detecting resistance to carbapenem, penicillin, and cephalosporin were merely 67.74%, 28.57%, and 46.15%, respectively, which significantly lower than our findings (Gan et al., 2024). Additionally, our study revealed discrepancies between tNGS and AST, where tNGS identified some AMR genes not detected by AST. This phenomenon observed in other studies as well (Gaston et al., 2022). That may be attributed to several factors, such as the low sensitivity of AST, non-pathogenic microorganisms carrying AMR genes, or heteroresistance within bacterial populations (Folkvardsen et al., 2013a; Folkvardsen et al., 2013b; Rinder et al., 2001; Wyres et al., 2014). By establishing new thresholds for tNGS, we significantly enhanced its consistency with AST, emphasizing the importance of tailored approaches for optimizing tNGS diagnostics. On the other hand, some antimicrobial resistant bacteria did not have their anticipated AMR genes found by tNGS, likely attributable to their low RPM. This observation underscores an inherent limitation of tNGS, as well as other genotypic approaches (Chiu and Miller, 2019).

The findings of this study underscore the potential of tNGS to revolutionize VAP diagnostics and management. Its high sensitivity, rapid turnaround time, and capability to identify AMR position it as a valuable tool for improving patient outcomes. However, limitations remain, including the impact of low-abundance pathogens on AMR detection and the inability to quantify pathogen load. Further research is needed to refine tNGS methodologies, explore its clinical impact on therapeutic decisions, and assess its integration into routine patient management workflows.

In conclusion, tNGS has outstanding performance in identifying pathogens and AMR in patients with VAP, particularly its high sensitivity. Given the notably rapid workflow of tNGS, it is particularly well-suited as a complement of traditional methods for managing critically ill patients. With further validation through large-scale, multi-center studies, tNGS holds promise for integration into routine clinical practice as a standard approach for infection detection.

## Data Availability

The datasets presented in this study can be found in online repositories. The names of the repository/repositories and accession number(s) can be found in the article/**Supplementary Material**.
