# Supplementary material for: Targeted next-generation sequencing for antimicrobial resistance detection in ventilator-associated pneumonia
Source: Front Cell Infect Microbiol. 2025 Jan 31;15:1526087. doi: 10.3389/fcimb.2025.1526087 (PMC11825505; doi:10.3389/fcimb.2025.1526087)
Supplement: Supplementary file 1 [file DataSheet1.docx]

Supplementary Material

# Supplementary Figures

**
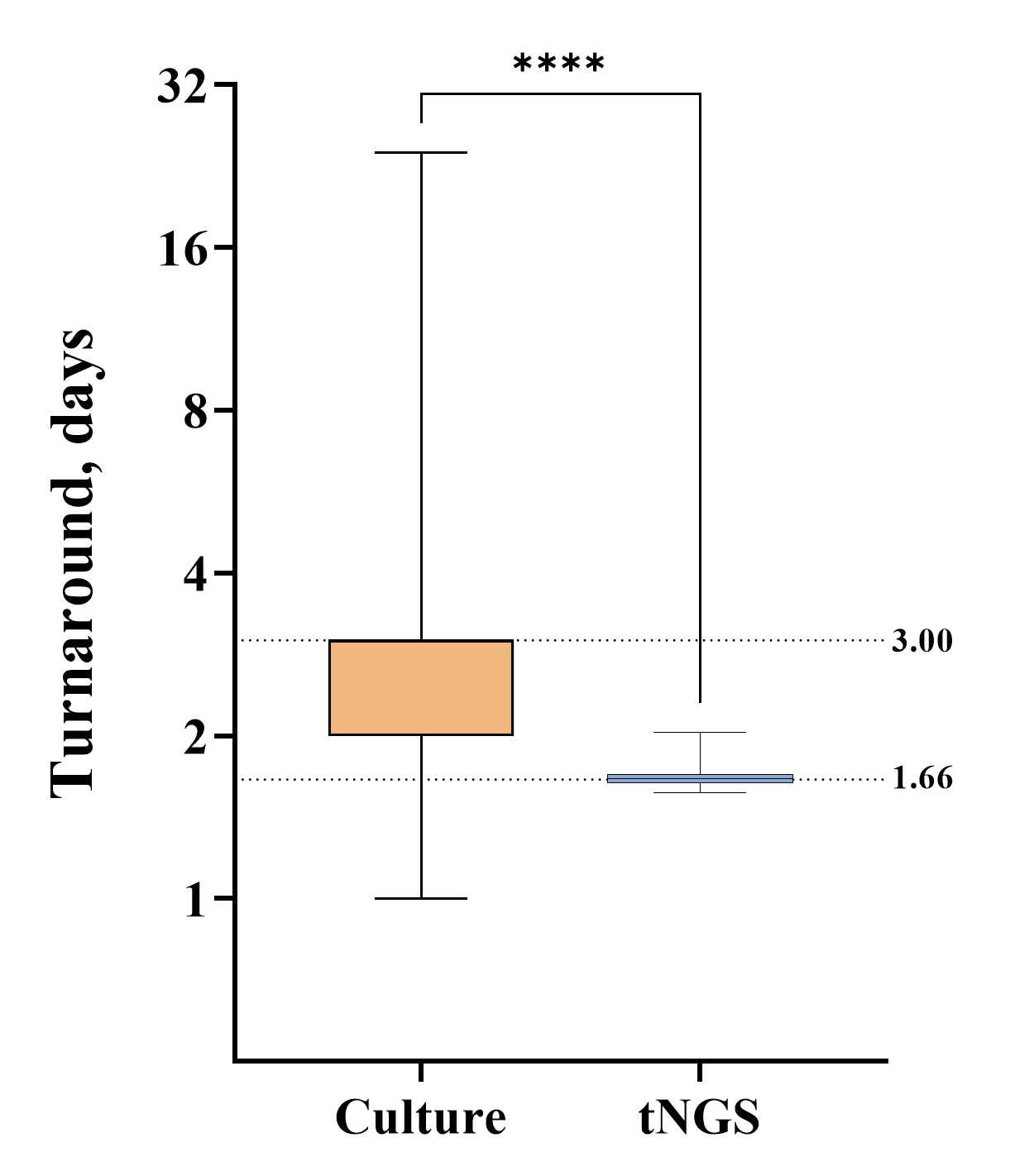
**

**Supplementary Figure 1.** Turnaround time of microbial culture and tNGS.

**
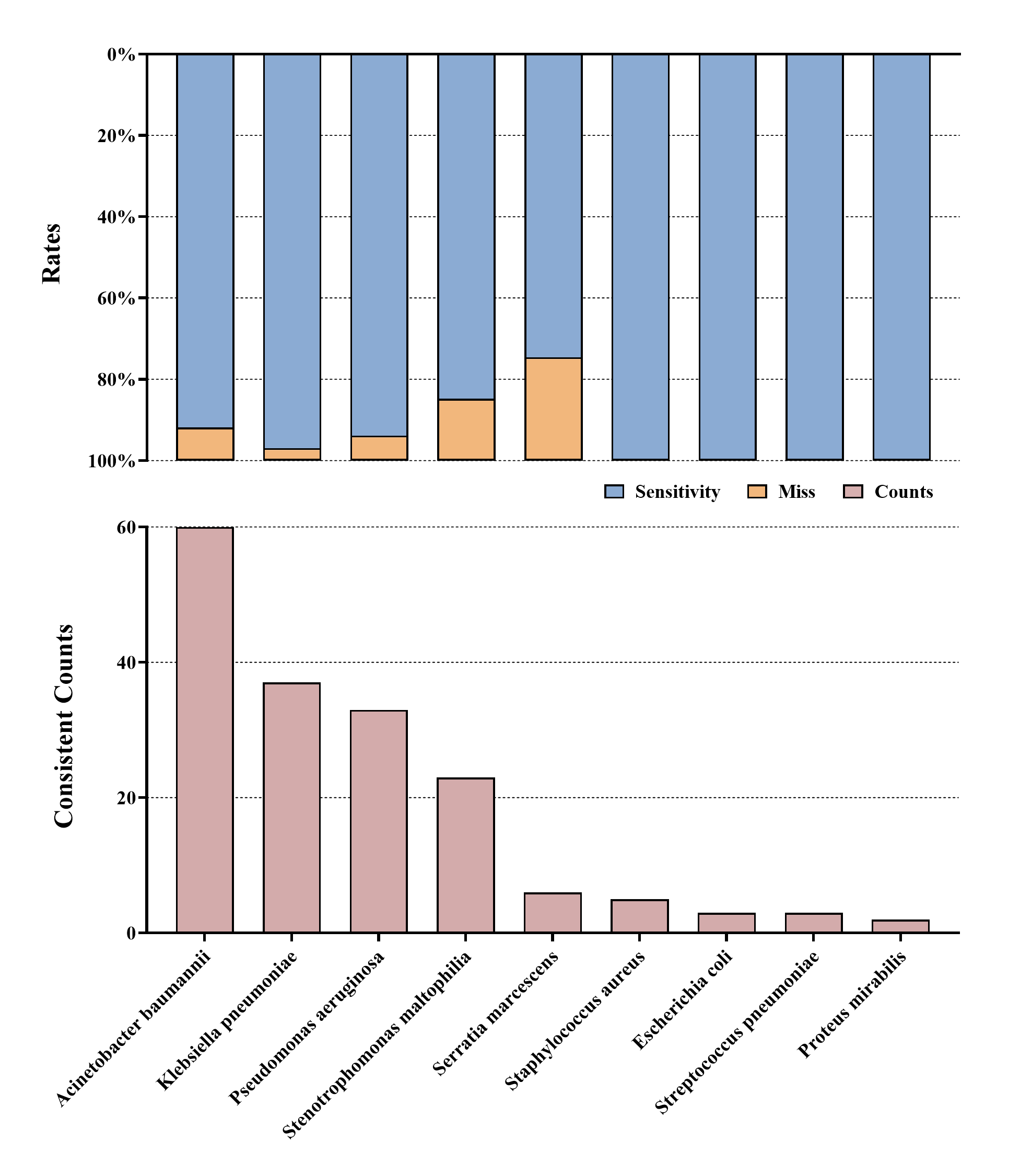
**

**Supplementary Figure 2.** Common pathogens reported consistently by tNGS and comprehensive clinical diagnosis.

**
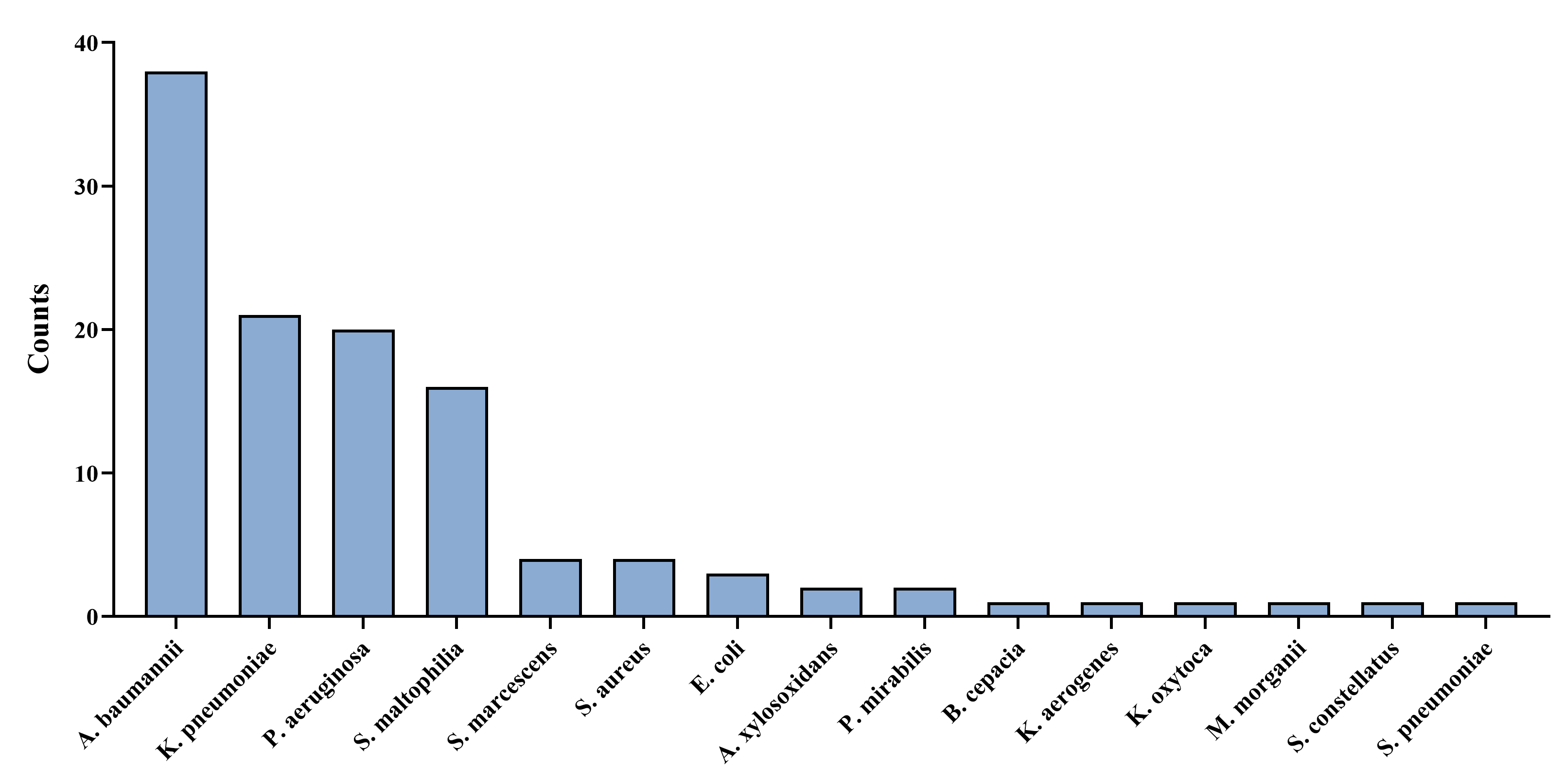
**

**Supplementary Figure 3.** Distribution of AMR bacteria reported consistently by tNGS and microbial culture.

# Supplementary Tables

**Supplementary Table 1.** Clinical characteristics of patients included.

| Characteristics | Patients |
| --- | --- |
| Age, years, median (Q1, Q3) | 68.0 (59.0, 74.8) |
| Gender, female, n (%) | 56 (28.1) |
| SOFA score, median (Q1, Q3) | 6.0 (4.0-9.0) |
| Mechanical ventilation duration, hours, median (Q1, Q3) | 288.0 (167.0, 480.0) |
| ICU stay duration, days, median (Q1, Q3) | 18.0 (11.0, 31.0) |
| Outcome, death, n (%) | 47 (23.6) |
| Dyspnea, n (%) | 122 (61.3) |
| Fever, n (%) | 102 (51.3) |
| Cough, n (%) | 27 (13.6) |
| WBC, 10^9/L, median (Q1, Q3) | 9.8 (7.4, 14.1) |
| Neutrophil ratio, %, median (Q1, Q3) | 86.5 (80.0, 91.0) |
| Lymphocyte ratio, %, median (Q1, Q3) | 7.0 (4.0, 11.0) |
| D-Dimer, mg/L, median (Q1, Q3) | 6.7 (2.2, 13.8) |
| CRP, mg/L, median (Q1, Q3) | 77.8 (33.9, 151.5) |
| PCT, ng/L, median (Q1, Q3) | 0.7 (0.2, 2.8) |

SOFA, sequential organ failure assessment; WBC, white blood cell; CRP, c-reactive protein; PCT, procalcitonin.
